# Targeting senescence to delay progression of multiple sclerosis

**DOI:** 10.1007/s00109-018-1686-x

**Published:** 2018-09-18

**Authors:** Wendy Oost, Nynke Talma, Jan F. Meilof, Jon D. Laman

**Affiliations:** 1 University of Groningen, Groningen, The Netherlands; 2 European Institute for the Biology of Ageing (ERIBA), University Medical Center Groningen, University of Groningen, Groningen, The Netherlands; 3 Department of Neuroscience, University Medical Center Groningen, University of Groningen, Groningen, The Netherlands; 4 Department of Neurology, Martini Hospital, Groningen, The Netherlands; 5 MS Center Noord Nederland (MSCNN), University Medical Center Groningen, University of Groningen, Groningen, the Netherlands

**Keywords:** Aging, Senolytics, Glia, Neurodegeneration, Inflammation, Autoimmunity

## Abstract

Multiple sclerosis (MS) is a chronic and often progressive, demyelinating disease of the central nervous system (CNS) white and gray matter and the single most common cause of disability in young adults. Age is one of the factors most strongly influencing the course of progression in MS. One of the hallmarks of aging is cellular senescence. The elimination of senescent cells with senolytics has very recently been shown to delay age-related dysfunction in animal models for other neurological diseases. In this review, the possible link between cellular senescence and the progression of MS is discussed, and the potential use of senolytics as a treatment for progressive MS is explored. Currently, there is no cure for MS and there are limited treatment options to slow the progression of MS. Current treatment is based on immunomodulatory approaches. Various cell types present in the CNS can become senescent and thus potentially contribute to MS disease progression. We propose that, after cellular senescence has indeed been shown to be directly implicated in disease progression, administration of senolytics should be tested as a potential therapeutic approach for the treatment of progressive MS.

## Introduction

Age is one of the most influential factors in MS progression [1, 2]. Several studies have shown that age affects disease progression of MS independently of initial disease pattern, disease duration, and gender [2, 3]. Aging can be defined as the time-dependent decline of functional capacity, which affects most living organisms [4]. The nine hallmarks that are generally considered to contribute to the aging process are genomic instability, telomere attrition, epigenetic alterations, loss of proteostasis, deregulated nutrient sensing, mitochondrial dysfunction, cellular senescence, stem cell exhaustion, and altered intercellular communication. These hallmarks are interconnected and contribute to aging and the development of age-related diseases [4]. Cellular senescence, one of the major hallmarks of the aging process, is a phenomenon by which cells go into irreversible growth arrest and become resistant to apoptosis. The number of senescent cells present in the human body increases with aging, which can have deleterious effects on the tissue microenvironment [5].

Several drugs have been approved for the treatment of relapsing-remitting phase of MS. Unfortunately, these drugs show little or no therapeutic effect in progressive MS. The first drug which was approved for the treatment of relapsing-remitting MS (RR-MS) was interferon-β_1_ (IFN-β_1_). To date, there are 13 FDA-approved drugs available for treatment of RR-MS. In general, these drugs act mainly by suppressing or altering the immune system. Also, these drugs have side effects, do not halt or reverse the disease, and most have limited long-term effectiveness [6]. The exception may be alemtuzumab for which durable efficiency was reported in people with RR-MS, including confirmed disability improvement [7]. However, it is unknown how long the drug effectiveness may last. Recently, ocrelizumab was approved as the first drug for treatment of primary progressive MS (PP-MS). Ocrelizumab is a humanized monoclonal antibody designed to selectively target CD20-positive B cells [8]. In this trial, a subset of people with PP-MS receiving ocrelizumab showed a moderate degree of slowing of disability accumulation compared to the placebo group [8]. In addition to suppression of ongoing inflammation, remyelination is essential in MS to restore saltatory conduction and axonal protection. Promotion of remyelination and/or inhibition of demyelination is critical to prevent further neuronal loss and cognitive decline observed in (progressive) MS [9]. Failure of remyelination is one of the pathologic hallmarks of progressive MS.

The relation of aging with disease progression in MS lends strong support to the hypothesis that progression could potentially be induced by increased cellular senescence in the CNS. Eliminating senescent cells delays age-related dysfunction in mouse models [10, 11]. Therefore, the aim of this review is to explore whether elimination of senescent cells could be a potential therapeutic strategy for delaying progression of MS. When cellular senescence is involved in MS disease progression, one could consider senolytics as a therapeutic treatment to delay progression. First, cellular senescence and its links with the progression of MS are discussed. Second, the concept of senolytics and the potential use of these drugs which specifically target senescent cells as a treatment for progressive MS will be discussed.

### Cellular senescence

Cellular senescence can be defined as an irreversible arrest of the cell cycle coupled to stereotyped phenotypic changes to decrease the risk for malignant transformation of the cell [5]. The term senescence was first introduced by Hayflick and Moorhead to describe the phenomenon of irreversible growth arrest in serially passaged human fibroblast culture, also known as replicative senescence [12]. Now, it is known that the senescence observed here was caused by telomere attrition [12, 13]. Cellular senescence can also be induced by many other stressors, including mitochondrial deterioration, oxidative stress, the expression of certain oncogenes, DNA damage, chromatin disruption, spindle stress, low expression of the mitotic spindle checkpoint protein budding uninhibited by benzimidazole-related 1 (BubR1), and other insults [14]. Senescence can be characterized by various markers, none of which is specific to senescent cells only and senescent cells may express only some of the markers used to characterize senescence. Major examples of these markers are G1 arrest (high expression of cell-cycle inhibitors p16^Ink4a^ and p21), high senescence-associated β-galactosidase (SA-β-gal) activity, altered epigenome, oxidative stress, DNA damage, and most importantly the senescence-associated secretory phenotype (SASP) as detailed in **Box 1** [5, 15].

**Table:**
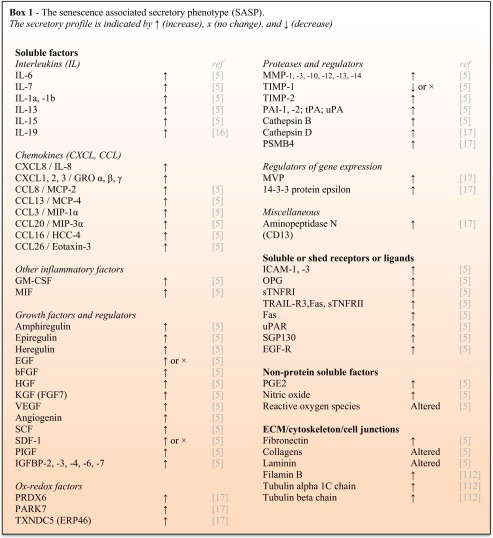

#### The secretory phenotype of senescent cells

One of the key characteristics that distinguish senescent cells from other non-proliferating cells is the SASP (**Box 1**). The SASP refers to the release of a wide array of pro-inflammatory cytokines and chemokines, tissue-damaging proteases, factors influencing stem- and progenitor cell function, and haemostatic factors and growth factors among other factors. The SASP factors can lead to the development of local and systemic pathogenic effects [5]. However, the influence of the SASP on the microenvironment can also be beneficial during embryonic development or in an acute setting of wound healing [18, 19].

The SASP factors can be subdivided into soluble signaling factors, soluble shed receptors or ligands, non-protein soluble factors, and insoluble factors (extracellular matrix (ECM)/cytoskeleton/cell junctions). Soluble signaling factors are the major components of the SASP and can be subdivided into interleukins, chemokines, other inflammatory factors, growth factors, ox-redox factors, proteases and their regulators, regulators of gene expression, and miscellaneous.

The most prominent SASP cytokine is IL-6, which is associated with senescence in various cell types such as mouse and human keratinocytes, melanocytes, monocytes, fibroblasts, and epithelial cells [20–22]. Senescent cells can also influence the tissue microenvironment by the secretion of non-protein soluble factors, such as reactive oxygen species (ROS) [23–25]. Moreover, senescent cells can have increased fibronectin expression as shown in prematurely aging fibroblasts in Werner Syndrome [26, 27]. Fibronectin is a major ECM glycoprotein of connective tissue, on cell surfaces and in plasma and other body fluids. The glycoprotein can bind to integrins and ECM components, such as collagen, and plays major roles in cell adhesion, growth, migration, and differentiation.

#### Mechanisms of tissue deterioration by cellular senescence

**Fig. 1:**
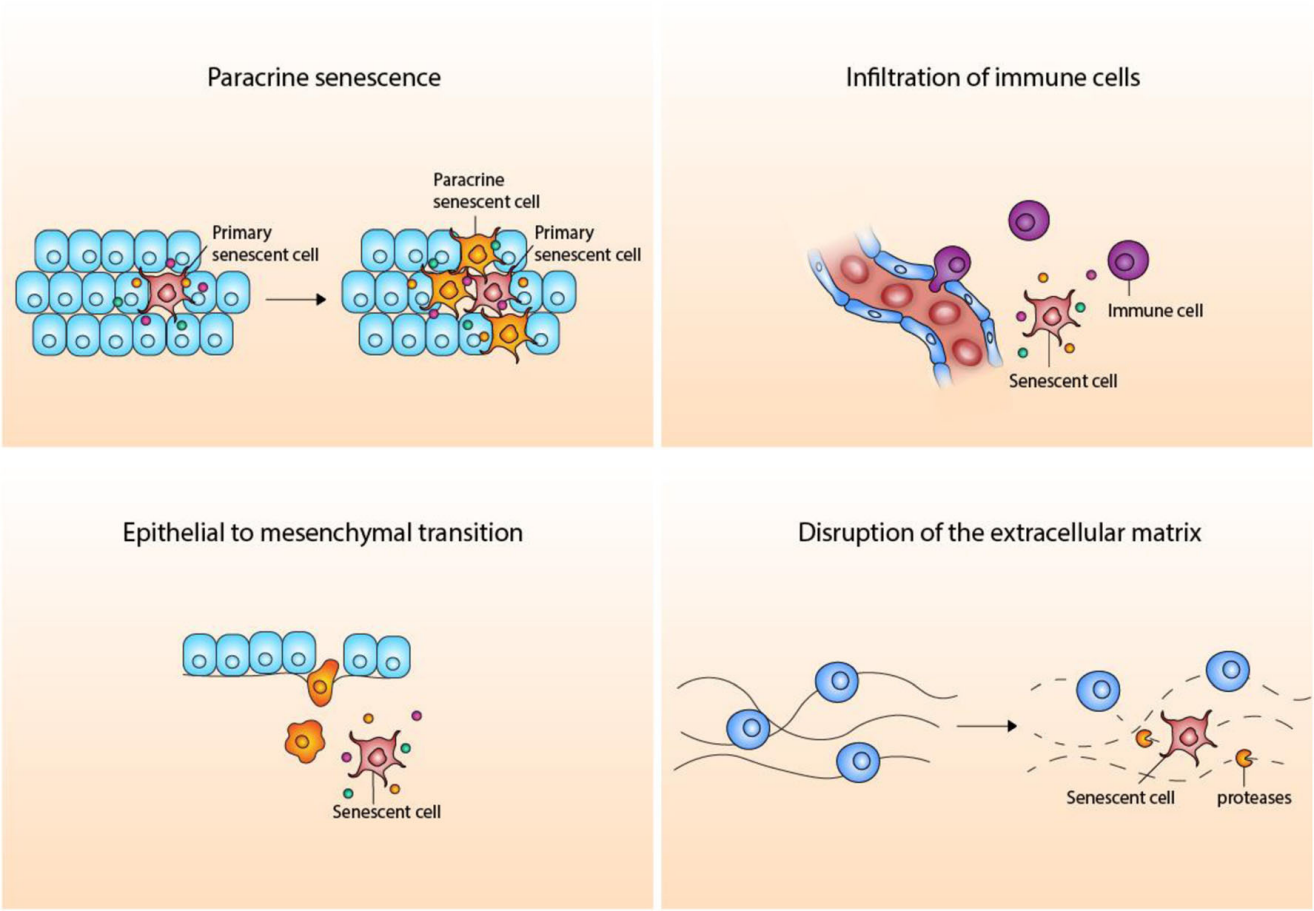
Mechanisms of tissue deterioration by cellular senescence. Cellular senescence can contribute to age-related tissue dysfunction by at least the following general mechanisms: paracrine senescence, stimulation of the infiltration of immune cells, disruption of the extracellular matrix, and by induction of epithelial to mesenchymal transition

Senescent cells are found in the affected tissues of patients with age-related diseases and are thought to promote age-related tissue dysfunction. The age-related diseases osteoarthritis, pulmonary fibrosis, diabetes, atherosclerosis, and Alzheimer’s disease are already thought to be connected with cellular senescence, suggesting that cellular senescence could be associated with their genesis and progression [28]. The SASP can contribute to age-related inflammatory diseases by causing a paracrine spread of cell dysfunction and tissue damage [29]. This can be induced by disruption of the stem cell niche, and therefore disruption of the tissue regeneration, by SASP factors [30, 31]. Furthermore, SASP proteases might also cause disruption of the extracellular matrix by cleavage of components of the tissue microenvironment [32]. Other SASP components, including IL-6 and IL-8, may induce epithelial-mesenchymal transition, stimulating tissue fibrosis and tumor metastasis [33, 34]. Senescent cells may also play a role in chronic tissue inflammation associated with the development of age-related diseases. Senescent cells accumulate in tissues manifesting age-related inflammatory pathologies and promote this inflammation through pro-inflammatory SASP factors. SASP factors IL-1β, TGFβ, and certain chemokine ligands may induce senescence in neighboring cells, sustaining and also exacerbating the previously mentioned mechanisms of tissue deterioration by increasing the number of senescent cells (Fig. 1) [35].

## Aging and cellular senescence in MS

MS is a chronic, often progressive, demyelinating disease of the CNS white and gray matter. Despite the fact that the disease course and symptomatology of MS are very heterogeneous from person to person, several disease subtypes can be recognized. The most common subtype is RR-MS, characterized by acute episodes of neurological deficits followed by periods of recovery. Aging seems to be a significant factor in MS disease progression. Those diagnosed with RR-MS have a 50% chance to transit to secondary progressive MS (SP-MS) within 10 years, and a 90% chance within 25 years. SP-MS is characterized by progressive decline, with or without relapses. In approximately 10–20% of the individuals that develop MS, the disease from the start slowly progresses over time and there are no (or occasionally minor) signs of remission after onset of the initial symptoms. This subtype is referred to as PP-MS. The histopathological hallmarks of MS are multifocal lesions with demyelination, oligodendrocyte death, axonal loss, and accumulation of blood-borne immune cells. The RR-MS course is characterized by inflammation followed by demyelination, adaptive immunity, activated astrocytes, disturbed blood-brain barrier (BBB) function, and (incomplete) remyelination. Conversely, SP-MS course is characterized by axonal degeneration, innate immunity, reactive astrocytes (gliosis), closed BBB, and hardly any remyelination [36].

The age distribution of MS is shifting to older age groups, from a peak prevalence of 50–54 years in 1984 to 55–59 years in 2004. People over the age of 65 who have been diagnosed with MS are more likely to have PP-MS or have an earlier transition to a SP-MS course, compared to younger people (under 65) [3, 37, 38]. MS disease progression can occur at varying rates between individuals, possibly due to varying underlying biological mechanisms often related to genetic and/or environmental factors [39, 40]. Importantly, increasing age has been shown to be a strong predictor for progression in MS, independent of the subtype and age of onset [38].

### Mechanisms of senescence-related MS disease progression

**Fig. 2:**
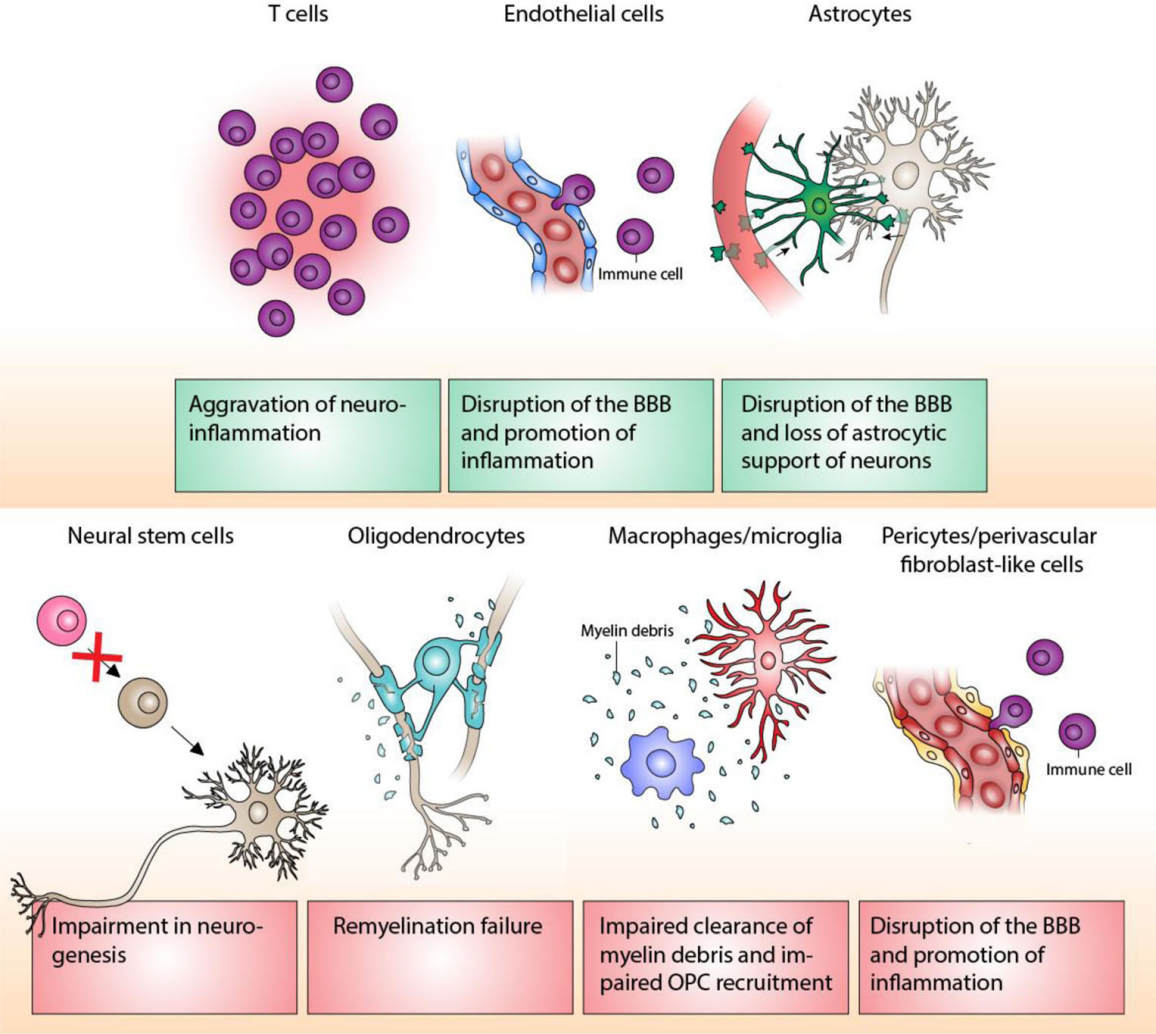
Potential mechanisms contributing to MS disease progression by senescence of different cell types. Different cell types could potentially become senescent and contribute to MS disease progression. The putative mechanisms are shown in the red and green boxes. Green boxes indicate that senescence of these cell types has been observed in vivo; red boxes indicate that senescence of these cell types has not yet been observed in vivo. Free-ware images of microglia, oligodendrocyte, astrocyte, and neuron are based on “cells of the CNS” [42]

It was recently shown that chronic demyelination observed in a cuprizone mouse model is associated with accelerated glial cell senescence in demyelinated lesions [41]. Moreover, inflammation, ROS, and fibronectin accumulation, which are also part of the SASP, are thought to play a role in the pathogenesis of MS. This suggests that the age-related disease progression observed in MS could potentially be functionally linked to cellular senescence. Here, we describe the mechanisms by which different senescent cell types could influence MS disease progression. An overview of these mechanisms is shown in Fig. 2.

#### Senescence of microglia and macrophages

The aging CNS shows decreased capacity for tissue repair, which could contribute to the progression of MS [43, 44]. One potential mechanism for this decreased repair response is immune-aging of microglia and macrophages [45]. Microglia are the resident immune cells of the CNS providing surveillance during homeostasis and against a wide variety of insults. This surveillance state is maintained by soluble molecules expressed in the CNS, such as transforming growth factor β, and ligands expressed on the surface of neurons, astrocytes, and oligodendrocytes [46]. Microglia are activated in MS, possibly promoting the infiltration of monocytes and lymphocytes into the CNS, changes in BBB integrity and secretion of inflammatory cytokines. Finally, this could promote to neuronal damage and death. Microglia also reactivate T cells, which enter the CNS from the periphery [47].

One of the main pathological differences between progressive and non-progressive MS is remyelination failure. Remyelination is essential for restoration of saltatory conduction and axonal protection [48]. Remyelination does occur in the early stages of the disease, but it declines as the disease progresses [44]. The expression of genes involved in the retinoid X receptor pathway is decreased in aging myelin-phagocytosing macrophages [49]. In addition, disruption of retinoid X receptor function in young macrophages results in aging-related decrease in myelin debris uptake [49]. Furthermore, retinoid X receptor agonists were able to partially restore myelin debris clearance. These results suggest that the retinoid X receptor pathway, which shows decreased activity in aging macrophages, plays a key role in remyelination due to its influence on myelin debris clearance [49, 50]. Moreover, aging microglia show decreased motility and cellular migration in response to tissue injury compared to young microglia [51]. Also, macrophages exhibit a decreased ability to produce a pro-inflammatory response, while microglia show an increased ability to produce a pro-inflammatory response [46, 52]. This increased microglial response is referred to as microglial priming and might lead to increased neuronal loss and accelerated progression in MS [53]. The age-associated delay in remyelination efficiency has been associated with reduction in macrophage/microglia recruitment in a toxin-induced demyelinating model [54]. The age-associated delay in remyelation can be explained by the decreased ability to resolve the inflammatory response initiated after myelin damage [55]. The breakdown of myelin results in the release of large amounts of cholesterol from the myelin [55]. Ingestion of myelin debris by macrophages induces an anti-inflammatory program [56]. Very recent mouse studies demonstrate large amounts of cholesterol overwhelm the efflux capacity of aged phagocytes, resulting in a phase transition of cholesterol into crystals and thereby inducing lysosomal rupture and NOD-like receptor family pyrin domain-containing protein 3 (NLRP3) inflammasome stimulation [55]. Thus, age-related defective cholesterol clearance limits remyelination.

Microglia and macrophages play a major role in the clearance of myelin debris and the recruitment of oligodendrocyte precursor cells (OPC) to the lesion site [57]. Aged microglia and macrophages show decreased phagocytosis and chemotaxis [58]. Decreased chemotaxis could result in impairment of the recruitment of endogenous OPC. The impairment in phagocytosis could result in impaired clearance of myelin debris and subsequent arrest of the differentiation of OPC [50]. The exact role of senescent microglia and macrophages in these processes is not known. There is some evidence supporting the induction of cellular senescence in microglia. In vitro experiments with the microglia-like cell line BV2 showed that these cells go into senescence after multiple inflammatory challenges indicating that this might also be possible in vivo [59]. The actual existence of senescent microglia and macrophages in vivo is yet to be confirmed.

#### Senescence of T cells

Besides microglia and macrophages, senescent brain-infiltrating T cells are likely critical in the progression of MS. Unlike the previously mentioned cell types, it has been shown that T cells can become senescent in vivo, as reflected by increased expression of CD57 and killer cell lectin like receptor G1 (KLRG1) on CD8^+^ T cells from aged individuals [60]. The numbers of senescent CD8^+^ T cells are increased in the aging brain and their increased pro-inflammatory cytokine production could aggravate the neuro-inflammation. This could worsen the cognitive function and drive progression of MS, since cytotoxic CD8^+^ T cells can drive neuronal damage [61]. CD8^+^ T cells are found abundantly in MS lesions and the number of cells correlates with the axonal damage rate in the lesions [62, 63]. Furthermore, all CNS cell types show increased MHC class I expression in the lesions [64]. This suggests that senescent, dysfunctional CD8^+^ T cells can target glial cells and neurons by direct recognition of the cell and/or myelin sheath via MHC class I or by excessive cytokine production, and therefore they likely play a role in the progression of MS.

#### Senescence of astrocytes

Aging can also influence the function of astrocytes, the most abundant cell type in the brain. Senescence of astrocytes may lead to changes in many astrocyte-regulated processes among which synaptic plasticity, metabolic balance, and BBB permeability. Senescent astrocytes have an increased expression of glial fibrillary acidic protein (GFAP) and vimentin filaments, increased expression of pro-inflammatory cytokines, and increased accumulation of proteotoxic aggregates [65]. GFAP levels in CSF of rodents and humans correlate with age and disease progression in MS, suggesting that senescent astrocytes might also contribute to disease progression [66, 67]. Senescent astrocytes could have a decreased capacity to support neurogenesis and to provide neuronal protection. GFAP/vimentin knockout mice have an increased cellular proliferation and neurogenesis [68]. Moreover, astrocytes without GFAP have an increased capacity to provide neuronal survival and neurite outgrowth compared to wild-type astrocytes [69]. Furthermore, senescent astrocytes have an increased expression of pro-inflammatory cytokines, such as IL-6, TNF-α, IL-1β, and prostaglandins, which negatively affect BBB function [70].

Astrocytes also have an important role in the neuron-glial crosstalk; they maintain metabolic and ion homeostasis of neurons, modulate synaptic transmission via glutamate, and they modulate neuronal activity [71, 72]. Therefore, senescence of astrocytes might promote neuronal dysfunction and degeneration, contributing to the progression of MS.

Glial scar formation, also referred to as gliosis, is effected by reactive astrocytes and it develops as the disease progresses [73]. Therefore, this process could be related to cellular senescence of astrocytes and other CNS cell types. Gliosis can have both beneficial and detrimental effects. The supposed beneficial effect is to physically isolate the damaged CNS areas to prevent spread of tissue destruction. Nonetheless, this process also has detrimental effects since it inhibits remyelination and axonal regeneration. The overproduction of FGF-2 and hyaluronan by astrocytes inhibits OPC differentiation and therefore remyelination. Also, the release of chondroitin sulphate proteoglycans (CSGP), ephrins (EPH), and their receptors, as well as myelin-associated inhibitors (MAI), inhibits remyelination and suppresses axonal growth [73].

Nonetheless, the role of senescent astrocytes in MS is not well-studied and the previously mentioned mechanisms by which senescent astrocytes could influence MS disease progression are not yet confirmed. Interestingly, it has been shown very recently that post-mortem Parkinson’s disease (PD) brain samples show increased astrocytic senescence and that astrocytic senescence could be induced by the herbicide paraquat both in vitro and in vivo [74]. Moreover, clearance of senescent cells by using a special PD mouse model that allows selective depletion without apparent off-target effects mitigates paraquat-induced neuropathology. Therefore, accumulation of senescent astrocytes might contribute to development of sporadic PD [74].

#### Senescence of endothelial cells

The senescence of endothelial cells lining the surface of blood vessels in the CNS is thought to contribute to disruption of BBB function. Impaired barrier integrity was observed in an in vitro BBB model, composed of senescent endothelial cells, pericytes, and astrocytes [75]. In vivo experiments with BubR1^H/H^ progeria mice showed impaired BBB integrity and increased senescence of endothelial cells and pericytes. Moreover, oxidative stress can induce endothelial senescence possibly by downregulation of sirtuin 6 (Sirt6), a regulator of endothelial cell senescence [76]. Radiation-induced senescence of endothelial cells results in the downregulation of a disintegrin and metalloprotease (ADAM) 10 [77]. This protein is the alpha-secretase that cleaves amyloid precursor protein (APP). This mechanism is considered important in preventing the formation of amyloid beta in Alzheimer’s disease (AD). In addition, ADAM10 can cleave many more proteins, including TNF-α and E-cadherin, and thereby promoting inflammation and affecting epithelial cell-cell adhesion [78, 79]. ADAM10 can also cleave the extracellular domain of the B cell-activating factor (BAFF)—a proliferation-inducing ligand (APRIL)—receptor transmembrane activator and calcium modulator and cyclophilin ligand interactor (TACI), releasing soluble TACI (sTACI) [80]. The BAFF-APRIL system is involved in the regulation of B cell homeostasis. sTACI levels are increased in CSF of MS patients and are thought to induce B cell accumulation and activation [80].

#### Senescence of pericytes and perivascular fibroblast-like cells

In addition to endothelial cells and astrocytes, pericytes contribute to the BBB. However, the physiological role of these cells is not well established [81]. Pericytes are essential in maintaining the BBB during brain aging and loss of pericytes leads to reduction in brain microcirculation and BBB breakdown [82]. Senescence of these cells might lead to impairment of their normal function and therefore contribution to neuro-inflammation and neurodegeneration. Recently, perivascular fibroblast-like cells were identified. These cells are located between the vessel wall and the astrocytic end-feet, and show resemblance to lung fibroblasts combined with epithelial (Lama1), endothelial (Cdh5), and mesothelial (Efemp1) markers [83]. Perivascular fibroblast-like cells could be the origin of pathological fibroblasts [84]. These pathological fibroblast cells are activated in experimental models of neuro-inflammation such as EAE and infection with the neurotropic hepatitis virus, as evidenced by rapid production of chemokine receptor 7 ligands [85, 86]. Fibroblast activation during chronic CNS inflammation contributes to the inflammatory response by recruitment of immune cells at sites of inflammation and secretion pro-inflammatory cytokines and survival factors to retain activated immune cells [87]. Senescence of vascular cells contributes to BBB disruption, as shown by impaired barrier integrity and tight junction structure in an in vitro BBB model constructed with senescent endothelial cells and pericytes [75].

#### Senescence of oligodendrocytes

OPC migrate toward the injured axon site after signaling by microglia or astrocytes. At the site, they must differentiate into mature oligodendrocytes (OLG) to be able to remyelinate the axon. Most OPC, which have a crucial role in remyelination, can avoid replicative senescence [88]. Nonetheless, there is evidence that OPC can become senescent [89]. Senescence of OLG might decrease remyelination capacity of demyelinated axons, which could lead to decrease in signaling ability and loss of protection of neurons and eventually neuronal cell death in MS.

#### Senescence of neurons

Senescence of neural stem cells could reduce neuronal neurogenesis, which can also contribute to the aging-associated progression of MS [90]. The expression of transcriptional regulator Hmga2 (high-mobility group AT-hook 2) in neural stem cells declines with age, resulting in an increased expression of p16^Ink4a^ and p19^Arf^ which can both induce cellular senescence [91].

SA-β-gal is commonly used biomarker of cell senescence and is found to be increased in the hippocampus of 24-month-old mice [92]. However, relatively high expression of SA-β-gal activity was also found in the hippocampus of 3-month-old mice, suggesting that SA-β-gal in neurons is not a unique marker of neuronal senescence [92]. DNA damage does not increase the number of SA-β-gal-positive neurons [92]. Moreover, sustained DNA damage of post-mitotic neurons can lead to the development of a p21-dependent senescent phenotype [93]. However, p21 expression did not show days-in-culture-dependent changes in cortical neurons [92]. These findings suggest that cell-cycle regulators associated with cellular senescence may not be relevant markers of senescence in post-mitotic neurons. Alternatively, repressor element 1-silencing transcription factor (REST) could possibly be used as a specific marker of neuronal aging in vitro [92]. Despite the controversy about senescence markers for neurons, overall, these results suggest that neurons indeed can become senescent. Senescence then can have direct effects on neuronal function and possibly MS disease progression.

In AD models, neuronal senescence is thought to be triggered by amyloid β and tau hyper-phosphorylation/accumulation. Neuronal senescence eventually might cause chronic neurodegeneration and cognitive impairment [94, 95].

#### Role of the SASP

Increased levels of pro-inflammatory molecules, secreted by senescent cells, can promote inflammation and might promote progression of MS. SASP factors secreted by senescent cells are also able to influence the extracellular matrix. The extracellular matrix is one of the factors regulating migration and proliferation of oligodendrocyte progenitor cells and their differentiation. For example, fibronectin promotes proliferation and reduces myelin-like membrane formation [96]. Fibronectin is upregulated in MS lesions and CNS parenchyma, affecting remyelination [97, 98]. Astrocytes generate fibronectin aggregates upon engagement with inflammatory mediators [99]. The pro-inflammatory factors of the SASP could therefore induce the generation of fibronectin aggregates. Senescent endothelial cells and fibroblasts have increased fibronectin expression [27]. The age-related increase of senescent cells in MS could therefore contribute to the increased fibronectin expression and progression of MS.

### Targeting senescent cells with senolytic drugs

Potential therapeutic strategies to prevent the deleterious effects caused by senescent cells are based on preventing formation of senescent cells, removal of senescent cells, and targeting the effects of senescent cells. Preventing formation of senescent cells requires interference with pathways leading to senescence. In addition, cellular senescence is a defense mechanism against cancer and therefore long-term interference with these pathways is likely to promote cancer [100]. Preventing or ameliorating the effects of the SASP could also be a potential therapeutic approach to decrease inflammation and cancer risk. However, this also inhibits the beneficial arm of the SASP. Elimination of senescent cells, on the other hand, has a larger potential to delay age-related degenerative pathologies [10]. Cancer risk would be reduced by activation of the tumor-suppressive pathway, leading to cellular senescence and, by removal of senescent cells, prevent malignant transformation of neighboring cells [10, 101]. Furthermore, elimination of senescent cells can be done intermittently which does not affect the formation of new senescent cells for purposes such as wound healing [19].

Different agents, including small molecules, peptides, and antibodies, called senolytics, are being developed to specifically remove senescent cells [102]. Senescent cells are resistant to apoptosis despite their own pro-apoptotic SASP factor release. Senolytics are directed against the pro-survival pathways of senescent cells, also referred to as senescent cell anti-apoptotic pathways (SCAP). These SCAP, responsible for the survival of senescent cells, were identified as the Achilles’ heel of senescent cells [103]. Both cellular senescence and mitochondrial dysfunction are the hallmarks of aging and are closely interlinked; upregulation of the SCAP is related to senescence-associated mitochondrial dysfunction (SAMD) and, at the same time, SAMD also drives and maintains cellular senescence [4, 104].

Thus far, six different SCAP are known: Bcl-2/Bcl-X_L_ family, PI3K/Akt/ROS protective/metabolic, p53/p21/serpine, ephrins/dependence receptors/tyrosine kinases, HIF-1α, and heat shock protein 90 (HSP-90) [103, 105]. A number of senolytic drugs are developed based on interference with these SCAP. Current senolytics are listed in **Box 2**. Senolytics can selectively induce apoptosis in senescent cells and can potentially be used in multiple age-related phenotypes. Recently, it has been shown that intermittent oral administration of the senolytic cocktail of dasatinib and quercetin decreased the number of naturally occurring senescent cells and alleviated physical dysfunction and increased survival in old mice [106].

**Table:**
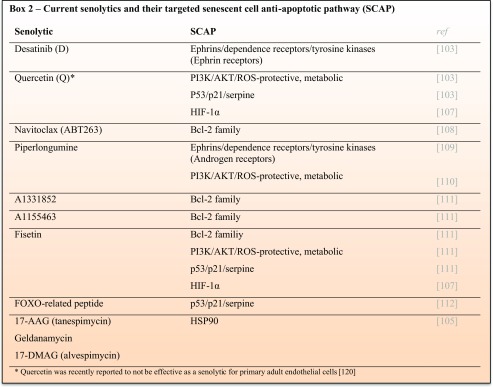

### Effects of cellular senescence in MS

There is initial evidence from animal models supporting the role of cellular senescence in the processes underlying disease progression in MS, for instance the increased cellular senescence observed in the cuprizone model [41]. This is a model in which young adult mice are fed with the copper chelator cuprizone, resulting in demyelination. This demyelination is caused by apoptotic cell death of oligodendrocytes, due to a disturbance in energy metabolism. This model has been associated with microglia and macrophage responses, but not with T cell activation and their recruitment into to the CNS [114]. In this model, they observed a 2.9-fold increase in senescent glial cell load in the corpus callosum as evidenced by SA-β-gal histochemistry at week 16.

Also in MS, there is some evidence for premature immunosenescence. One of the characteristics of immunosenescence is the expansion of CD4^+^ CD28^−^ T cells. These cells accumulate in MS lesions [115–117]. It has also been suggested that senescent CD8^+^ T cells could contribute to disease progression but the evidence is less clear [118].

The occurrence of other effects of cellular senescence in MS has only been hypothesized and has not been confirmed yet. For example, the chronic secretion of ROS generated by senescent cells and inflammatory cells as they attack myelin might cause a spread of demyelination. This can at least partly explain why cortical demyelination is found in patients suffering from progressive MS, but not in acute MS [119]. Another SASP factor that could contribute to MS disease progression is the ECM glycoprotein fibronectin. Fibronectin contributes to remyelination failure and increased fibronectin expression by senescent cells and can therefore enhance this phenomenon [97, 98]. Furthermore, the pro-inflammatory cytokines secreted by senescent cells can directly influence the surrounding brain tissue, which might drive neurodegeneration. Also, inflammatory mediators are thought to induce increased fibronectin expression by astrocytes [99]. Moreover, the age-related iron accumulation observed in progressive MS patients is likely to be a consequence of cellular senescence [36, 120]. In conclusion, senescence of cells present in the CNS in MS could contribute to MS disease progression, but more mechanistic research is necessary to support this hypothesis.

## Conclusions and future outlook

From our review we conclude that there is ample evidence to warrant further studies into the possible link between cellular senescence and progression of MS. Future in vitro studies on the different cell types present in the CNS can elucidate the mechanisms through which these cells become senescent and if the senescent phenotype alters their role in important processes such as myelin debris clearance, remyelination, and axonal protection. The results of these studies can be used to guide a focused search for senescent cells in MS lesions in post-mortem brain tissue from different MS subtypes. This will further strengthen the link between cellular senescence and disease progression in MS. The final preclinical step would be to test senolytic treatment protocols using in vitro (e.g., brain-on-a-chip) and in vivo (EAE, cuprizone) models of demyelination and MS.

Adding senolytic treatment to effective immunomodulatory and remyelination promoting therapy could result in a treatment strategy which can limit further disability accrual in patients with MS and thus have great impact on the prognosis for people with MS.

## Abbreviations

AD: Alzheimer’s disease
ADAM: A disintegrin and metalloprotease
APP: Amyloid precursor protein
APRIL: A proliferation-inducing ligand
BAFF: B cell-activating factor
BBB: Blood-brain barrier
BubR1: Budding uninhibited by benzimidazole-related 1
CCL: CC chemokine ligand
CD: Cluster of differentiation
CNS: Central nervous system
CSF: Cerebrospinal fluid
CSGP: Chondroitin sulphate proteoglycans
CXCL: Chemokine CXC motif ligand
EAE: Experimental autoimmune encephalomyelitis
ECM: Extracellular matrix
EGF: Endothelial growth factor
EPH: Ephrin
ERP: Endoplasmic reticulum protein
FGF: Fibroblast growth factor
GFAP: Glial fibrillary acidic protein
GM-CSF: Granulocyte-macrophage colony-stimulating factor
GRO: Growth regulated oncogene
HCC: Hepatocellular carcinoma-associated protein
HGF: Hepatocyte growth factor
Hmga2: High-mobility group AT-hook 2
HSP: Heat shock protein
ICAM: Intercellular adhesion molecule
IGF: Insulin-like growth factor
IGFBP: IGF binding protein
IL: Interleukin
KGF: Keratinocyte growth factor
KLRG1: Killer cell lectin like receptor G1
MAI: Myelin-associated inhibitors
MCP: Membrane cofactor protein
MHC: Major histocompatibility complex
MIF: Macrophage migration inhibitory factor
MIP: Macrophage inflammatory protein
MMP: Matrix metalloproteinases
MVP: Major vault protein
MS: Multiple sclerosis
NLRP3: NOD-like receptor family pyrin domain-containing protein 3
OLG: Oligodendrocyte
OPC: Oligodendrocyte precursor cells
OPG: Osteoprotegerin
PAI: Plasminogen activator inhibitor
PARK: Protein deglycase DJ-1
PD: Parkinson’s disease
PDH: Pyruvate dehydroxygenase
PDK1: Pyruvate dehydrogenase kinase 1
PDP2: Pyruvate dehyrogenase phosphatase catalytic subunit 2
PGE2: Prostaglandin E2
PI3K: Phosphatidylinositol 3-kinase
PIGF: Placental growth factor
PP-MS: Primary progressive MS
PRDX: Peroxiredoxin
PSMB: Proteasome subunit beta
REST: Repressor element 1-silencing transcription factor
ROS: Reactive oxygen species
RR-MS: Relapsing-remitting MS
SA-β-gal: Senescence-associated β-galactosidase
SAMD: Senescence-associated mitochondrial dysfunction
SASP: Senescence-associated secretory phenotype
SCAP: Senescent cell anti-apoptotic pathway
SCF: Stem cell factor
SDF: Stromal cell-derived factor
SGP: Soluble glycoprotein
Sirt6: Sirtuin 6
SP-MS: Secondary progressive MS
sTNFR: Soluble tumor necrosis factor receptor
TACI: Transmembrane activator and calcium modulator and cyclophilin ligand interactor
TGFβ: Transforming growth factor β
TIMP: Tissue inhibitor of metalloproteinases
TNF-α: Tumor necrosis factor α
TRAIL: Tumor necrosis factor-related apoptosis-inducing ligand
TXNDC: Thioredoxin domain containing
tPA: Tissue-plasminogen activator
uPA: Urokinase-type plasminogen activator
uPAR: uPA receptor
VEGF: Vascular endothelial growth factor
